# En bloc corpectomy for late gastrointestinal stromal tumor metastasis: a case report and review of the literature

**DOI:** 10.1186/s13256-018-1844-3

**Published:** 2018-10-16

**Authors:** Takaki Shimizu, Hideki Murakami, Apiruk Sangsin, Satoru Demura, Satoshi Kato, Kazuya Shinmura, Noriaki Yokogawa, Norihiro Oku, Ryo Kitagawa, Hiroyuki Tsuchiya

**Affiliations:** Department of Orthopaedic Surgery, Graduate School of Medical Sciences, Kanazawa University, 13-1 Takara-machi, Kanazawa, 920-8641 Japan

**Keywords:** Spinal metastasectomy, Gastrointestinal stromal tumor, Spinal metastases, En bloc corpectomy

## Abstract

**Background:**

Spinal metastases of gastrointestinal stromal tumors are rare; however, the incidence has been increasing since the introduction of tyrosine kinase inhibitors, which have improved overall survival. Due to the rarity of cases, there are no treatment guidelines for spinal metastases of gastrointestinal stromal tumors. We describe a patient who underwent spinal metastasectomy for a rectal gastrointestinal stromal tumor; we further provide a review of all cases of gastrointestinal stromal tumors with spinal metastases.

**Case presentation:**

A 51-year-old Japanese man who had undergone resection for a rectal gastrointestinal stromal tumor was diagnosed with L3 vertebral metastasis 10 years after surgery. As there were no metastases to vital organs, an en bloc corpectomy of the L3 vertebral body, using bilateral retroperitoneal approaches, was performed to achieve local cure and to prevent neural compression. A 3-year follow-up showed no local recurrence or new metastases; he had full neurological function.

**Conclusions:**

Spinal metastasectomy can be an effective treatment for solitary spinal metastases of gastrointestinal stromal tumors.

## Background

Gastrointestinal stromal tumors (GISTs) are the most common mesenchymal tumors of the digestive tract and are derived from the interstitial cells of Cajal [1]. Spinal metastases of GISTs are rare; however, their incidence has been increasing since the introduction of tyrosine kinase inhibitors (TKIs), which have improved overall survival [2]. We describe a case of L3 metastasis 10 years after rectal GIST resection in a 51-year-old man, along with a literature review.

This is the first report of radical metastasectomy for solitary spinal metastasis of GIST.

## Case presentation

### History and clinical evaluation

A 51-year-old Japanese man who was born and raised in Japan and did not have any underlying medical condition presented with a rectal mass. He underwent endoscopic biopsy of the lesion, and the histopathological report showed spindle-shaped tumor cells with mild cytological atypia. Immunohistochemical analysis revealed that the tumor was positive for CD117 (c-kit) and CD34, leading to the diagnosis of a rectal GIST. He underwent colectomy after shrinking the tumor with 400 mg/day of imatinib for 9 months. After completion of treatment, annual follow-up computed tomography (CT) scans showed no local recurrence or distant internal organ metastases. His postoperative course was good and he could continue working as a judo therapist. There was nothing notable in his medical history, except for the rectal GIST, and he did not take any medication after surgery. His family history was uneventful and he did not smoke tobacco or drink alcohol.

**Fig. 1:**
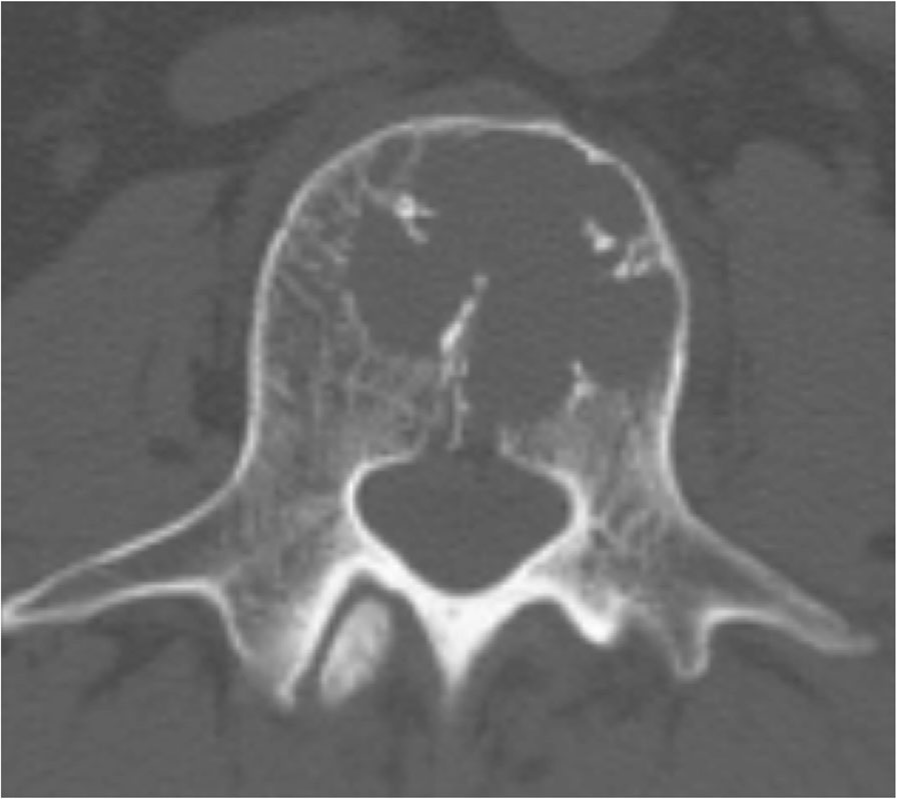
Preoperative computed tomography scan reveals osteolytic lesions in the anterior column of L3 vertebra

Although he was asymptomatic, a CT scan 10 years after surgery revealed a destructive osteolytic lesion in the L3 vertebral body (Fig. 1). CT-guided biopsy confirmed the lesions to be GIST metastases. His vital signs were stable with blood pressure 128/64, pulse rate 68 beats/minute, and temperature 36.3 °C. Sensations were normal in both lower limbs. His muscle strength was grade M5 throughout (Medical Research Council Scale of Muscle Strength), and deep tendon reflexes were normal. Laboratory testing showed a normal complete blood count (CBC). His liver and renal functions were sufficient, and the electrolytes were normal. As there were no metastases in vital organs and the tumor was located only in the anterior column of the vertebra, en bloc corpectomy of the L3 vertebral body was performed to provide local cure of the tumor and to prevent devastating sequelae of neural compression.

### Surgery

**Fig. 2:**
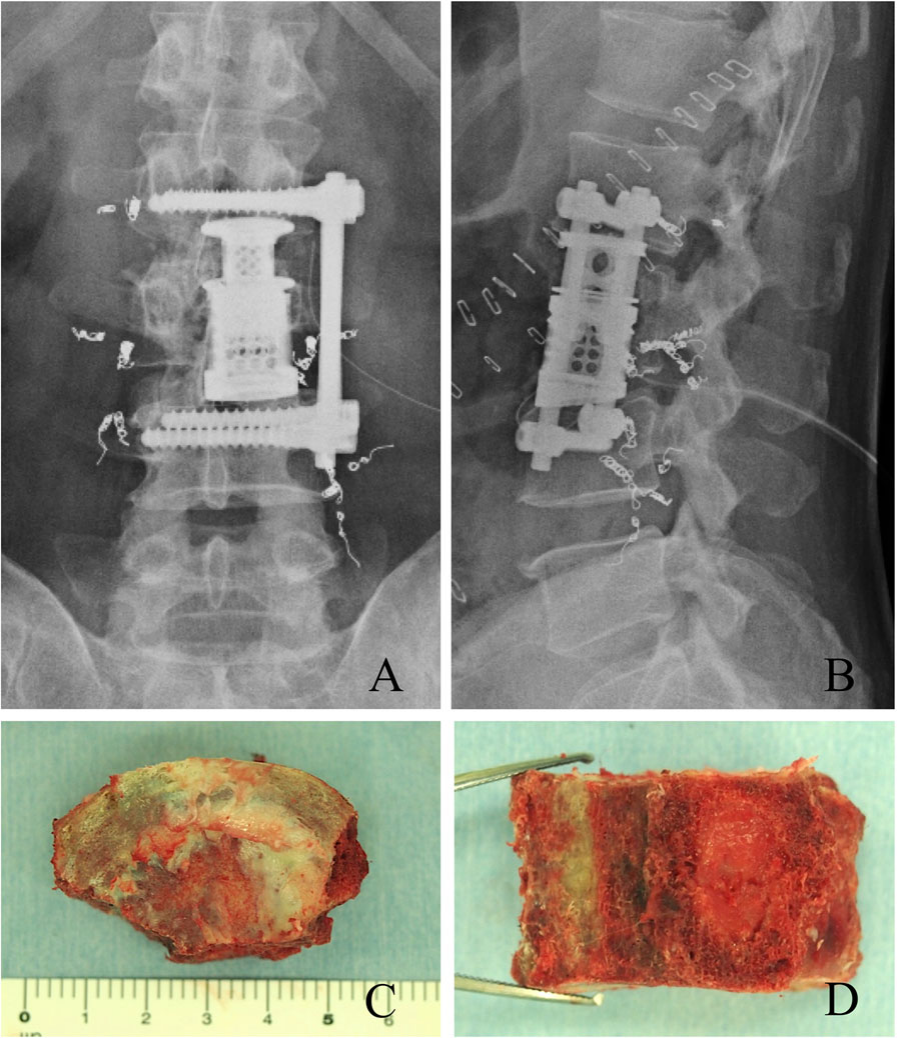
Frontal (**a**) and lateral (**b**) view of postoperative radiographs. Top view (**c**) and a cross-sectional view (**d**) of the resected L3 vertebral body

En bloc corpectomy via bilateral anterolateral retroperitoneal approaches was performed. Major vascular structures and the psoas muscles were retracted to expose the vertebral body and adjacent discs. The L3 vertebral body was cut off from the posterior elements using high-speed drills and chisels, and was removed en bloc. A titanium cage, with an autologous bone graft inside, was placed into the vertebral defect, and the spinal reconstruction procedure was finalized using screws and rods (Fig. 2).

### Postoperative course

The pathological finding of the resected vertebral body was consistent with GIST metastasis, and the surgical margin was negative. Laboratory testing 3 days after the surgery showed slightly low hemoglobin (127 g/L, reference range 135–170 g/L) and a C-reactive protein (CRP) level of 114 mg/L (reference range < 3 mg/L). His liver enzymes were elevated: alanine aminotransferase (ALT) 112 U/L (reference range < 35 U/L) and aspartate aminotransferase (AST) 40 U/L (reference range < 35 U/L). CRP, ALT, and AST levels completely normalized within 1 month after surgery. In repeated laboratory tests, CBC, CRP, and liver enzymes remained normal until the final follow-up. At the 36-month follow-up, no local recurrence, new metastasis, or instrumentation failure was observed on CT or magnetic resonance imaging. He had full neurological functions without any limitation of daily activities.

## Discussion and conclusions

This is a case report of metastasectomy performed for spinal metastasis, which appeared 10 years after rectal GIST surgery, with a good postoperative course. The unique points in this case are that the metastasis occurred after a very long period of time, and this is the first report of radical surgery for spinal metastasis of GIST.

GISTs are spindle, epithelioid, or pleomorphic mesenchymal tumors of the digestive tract that express CD117 (c-kit) [1]. GISTs are characterized by a gain-of-function mutation in *KIT* (78%) or *PDGFRA* (6%) genes [3]. Even in the era of TKIs, surgical resection remains the major treatment strategy for primary GISTs. However, local recurrence and metastasis typically occur [4, 5], and 40% of patients develop liver metastasis (65%) or peritoneal metastasis (50%) [6].

Bone metastases of GISTs, including the spine, are rare and only 12 cases with spinal metastases have been described to date (Table 1). However, spinal metastases in GISTs are now encountered more frequently than in the past because of an improvement in overall survival since the use of TKIs [2]. In most cases, spinal metastases of GISTs occur early in the course of the disease. Four (33%) of 12 reported cases had spinal metastases at initial presentation [2, 7, 8], while the others showed spinal metastases after a median time of 20.5 (4–84) months [2, 9, 10]. All the previously reported cases had spinal metastases associated with an uncontrollable primary tumor [8] or liver metastasis [2, 7, 9–11]. However, our patient, who underwent complete resection of the primary tumor, developed solitary metastasis of the L3 vertebral body 10 years after surgery, which was the latest spinal metastasis among past reports. Similar delayed metastasis may become more prevalent as TKI extends the survival time of patients with GIST.

**Table 1:** Clinical data for 12 cases of gastrointestinal stromal tumor with spinal metastases

Patient	Primary tumor location	Location of metastases/invasion	Spinal metastases treatment (aside from TKIs)	Reference
1	Small intestine	L, S, B	Radiotherapy + zoledronic acid	[2]
2	Small intestine	L, S, B	Radiotherapy + zoledronic acid	[2]
3	Stomach	L, V, B	Zoledronic acid	[2]
4	Sigmoid colon	L, V, B	–	[10]
5	Esophagus	V, M	Posterior decompression and instrumentation	[8]
6	Stomach	L, V, B, S, ST, LN, P	N/A	[11]
7	Rectum	L, V, B, P	N/A	[11]
8	Stomach	L, V, B, P	N/A	[11]
9	Stomach	L, V, B, P	N/A	[11]
10	Small intestine	L, V, B	Radiotherapy + zoledronic acid	[9]
11	Small intestine	L, V	Radiotherapy + zoledronic acid	[9]
12	Stomach	L, V, B	–	[7]
13	Rectum	V	En bloc corpectomy	Current report

*B* bone metastases aside from vertebrae, *L* liver, *LN* lymph node, *M* mediastinum, *N/A* not available, *P* peritoneum, *S* spleen, *ST* soft tissue, *TKI* tyrosine kinase inhibitor, *V* vertebrae

Few data are available in the literature on the treatment of spinal metastases in GISTs. Previously, most cases of spinal metastases were treated non-surgically with TKIs with or without zoledronic acid. Pain reduction and disease stabilization were observed in some cases, although the mechanism remains unclear [2, 7, 9, 11]. However, some cases may also show disease progression and drug resistance [2]. Spinal metastases of GISTs cause neurological deficit due to neural compression [8, 11], which can lead to deterioration in performance status or survival. In such cases, posterior decompression and instrumentation were performed for the treatment of spinal metastases [8]; however, they only had a palliative role, reducing pain or preventing pathologic fractures and other skeletal-related events. Unlike past reports, this case was a solitary metastasis and owing to unpredictable responses to TKIs and the controversial role of radiotherapy and chemotherapy in GISTs [12], metastasectomy using en bloc corpectomy was performed to avoid complications such as neural compression and obtain local cure. From an oncological point of view, en bloc resection of the tumor is mandatory for local control and recurrence prevention. In this case, en bloc corpectomy was performed as the tumor was confined to the vertebral body. However, a metastatic spinal tumor can often involve the pedicle and extend to the posterior element; total en bloc spondylectomy should be performed in such cases [13]. Suzuki *et al.* reported the case of two patients who underwent complete resection of bone metastases (other than the spine) of GIST and benefitted from long-term disease-free survival [14]. Although our study had a short-term follow-up, based on a satisfactory oncological outcome with preserved performance status, spinal metastasectomy can be an effective treatment for GISTs with solitary spinal metastases. However, the effect of spinal metastasectomy on various parameters of survival needs to be investigated in detail.

In conclusion, although the incidence of spinal metastasis of GISTs is currently low, it could become more prevalent because of longer patient survival after TKI therapy, which offers sufficient time for the occurrence of metastasis. The possibility of late metastasis, such as in the present case, should be acknowledged and more attention should be paid to the diagnosis of spinal metastases of GISTs. From the satisfactory outcome of this case, spinal metastasectomy can be an effective treatment for solitary spinal metastasis of GISTs. Furthermore, more case series should be extensively studied to establish surveillance protocols and treatment guidelines.

## Abbreviations

ALT: Alanine aminotransferase
AST: Aspartate aminotransferase
CBC: Complete blood count
CRP: C-reactive protein
CT: Computed tomography
GISTs: Gastrointestinal stromal tumors
TKIs: Tyrosine kinase inhibitors
